# A revised action spectrum for vitamin D synthesis by suberythemal UV radiation exposure in humans in vivo

**DOI:** 10.1073/pnas.2015867118

**Published:** 2021-09-27

**Authors:** Antony R. Young, Kylie A. Morgan, Graham I. Harrison, Karl P. Lawrence, Bibi Petersen, Hans Christian Wulf, Peter A. Philipsen

**Affiliations:** ^a^St. John’s Institute of Dermatology, School of Basic and Biomedical Sciences, Faculty of Life Sciences and Medicine, King’s College London, London SE1 9RT, United Kingdom;; ^b^Global Medical Affairs, LEO Pharma, 2750 Ballerup, Denmark;; ^c^Department of Dermatology D92, Copenhagen University Hospital - Bispebjerg, DK-2400 Copenhagen, Denmark

**Keywords:** vitamin D, action spectum, UVR, human skin

## Abstract

Action spectra are important biological weighting functions for risk/benefit analyses of ultraviolet (UV) radiation (UVR) exposure. One important human benefit of exposure to terrestrial solar UVB radiation (∼295 to 315 nm) is the cutaneous synthesis of vitamin D_3_ that is initiated by the photoconversion of 7-dehydrocholesterol to previtamin D_3_. An action spectrum for this process that is followed by other nonphotochemical steps to achieve biologically active vitamin D_3_ has been established from ex vivo data and is widely used, although its validity has been questioned. We tested this action spectrum in vivo by full- or partial-body suberythemal irradiation of 75 healthy young volunteers with five different polychromatic UVR spectra on five serial occasions. Serum 25-hydroxyvitamin D_3_ [25(OH)D_3_] levels, as the most accurate measure of vitamin D_3_ status, were assessed before, during, and after the exposures. These were then used to generate linear dose–response curves that were different for each UVR spectrum. It was established that the previtamin D_3_ action spectrum was not valid when related to the serum 25(OH)D_3_ levels, as weighting the UVR doses with this action spectrum did not result in a common regression line unless it was adjusted by a blue shift, with 5 nm giving the best fit. Such a blue shift is in accord with the published in vitro action spectra for vitamin D_3_ synthesis. Thus, calculations regarding the risk (typically erythema) versus the benefit of exposure to solar UVR based on the ex vivo previtamin D_3_ action spectrum require revision.

## Significance

Solar UV radiation (UVR) causes sunburn but initiates the first step of vitamin D synthesis, which is the formation of previtamin D_3_ (pre-D_3_) in skin. The gold standard for assessing vitamin D is serum 25-hydroxyvitamin D_3_ [25(OH)D_3_]. Public health advice for optimal solar exposure requires UVR wavelength-dependence (action spectrum) data on risks and benefits. An action spectrum for pre-D_3_ in human ex vivo skin was established over 30 y ago, but its validity has been questioned. We tested this action spectrum in healthy volunteers using serum 25(OH)D_3_ as the endpoint. Our analysis shows that the pre-D_3_ action spectrum can be improved with a systematic correction. This will result in better risk–benefit calculations for public health advice on solar exposure.

Exposure to solar ultraviolet (UV) radiation (UVR: ∼295 to 400 nm) has well-established adverse effects on human health. Recent research suggests that sunlight may have several benefits, including the reduction of blood pressure (1), other than vitamin D_3_ synthesis. Reliable public health advice on optimal solar exposure to obtain vitamin D, yet mitigate risk, requires diverse expertise apart from the vitamin D community. This includes photobiologists, dermatologists, epidemiologists, climatologists, atmospheric and UVR measurement physicists, mathematical modelers, and behavior and public health scientists.

7-Dehydrocholesterol (7-DHC), primarily in the epidermis, is photo converted to previtamin D_3_. This is thermally isomerized to vitamin D_3_ (cholecalciferol), which enters the blood stream and undergoes two hydroxylations to become the active circulating hormone (calcitriol, 1α,25(OH)_2_D_3_). The skin has enzymic capacity for both hydroxylations (2). Vitamin D may be obtained by diet (e.g., oily fish) and supplements, but terrestrial solar UVB (∼295 to 315 nm) radiation is the main source (3). The benefits of vitamin D for musculoskeletal health are established, but other health benefits are controversial (4–6).

Solar UVR exposure causes sunburn (erythema), skin cancer, and photoaging. Skin cancer is of greatest concern because its incidence is increasing in susceptible fair-skinned populations in many countries. Erythema is useful as a measure of personal UVR sensitivity when determined by the minimal erythema dose (MED) that is widely used in clinical and experimental photobiology. The MED is not suitable to measure population UVR exposure, and the standard erythema dose (SED) is preferred. This is a mathematical construct derived from the Commission Internationale l’Éclairage (CIE) wavelength-dependence (action spectrum) for erythema (7); in other words, one SED is an erythemally weighted dose (100 J/m^2^) of a given UVR emission spectrum that is independent of personal UVR sensitivity and UVR spectrum. One MED, on previously unexposed buttock skin, is about three SED in a fair-skinned type I/II person (8). Solar UVB is orders of magnitude more erythemogenic than UVA (315 to 400 nm) radiation, which means that its small terrestrial quantity (<5% total UVR) has a disproportionately large effect. The influence of the ozone layer means that UVB irradiance is more heavily dependent than UVA on solar zenith angle (SZA) that varies with latitude, season, and time of day. Thus, the relative erythemally effective energies (EEE) of solar UVB and UVA vary with the height of the sun. The action spectra for epidermal DNA photodamage (cyclobutane pyrimidine dimers) (9), keratinocyte cancers of the skin (10), and photoaging (11, 12) are broadly similar to that for erythema, which means that SED may be a useful spectral risk indicator for these endpoints. However, it should be noted that SED is unlikely to be a good indicator of UVA-induced oxidatively generated damage to a range of biomolecules, including nucleic acids, that may play a role in malignant melanoma that is the most dangerous type of skin cancer (13).

**Fig. 1.**
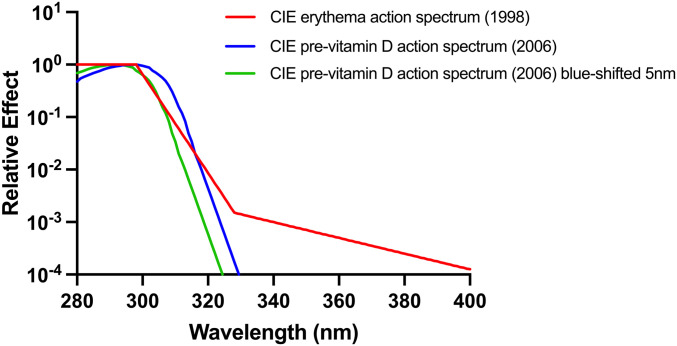
CIE action spectra for human erythema (7) and the cutaneous synthesis of previtamin D_3_ (14). The effect of a 5-nm blue shift on the previtamin D_3_ action spectrum is also shown.

The CIE has issued an action spectrum for previtamin D_3_ synthesis (14) that, along with the CIE erythema action spectrum (7), has been used extensively as a weighting function in risk–benefit analyses for solar UVR exposure (15, 16). Both CIE spectra have also been used to determine an optimum sunscreen absorption spectrum for vitamin D synthesis (17). In other words, calculations can be made of EEE and vitamin D effective energy under given solar exposure conditions and used for public health guidance. The CIE action spectra for erythema and previtamin D_3_ production are shown in Fig. 1. The validity of the CIE previtamin D_3_ action spectrum has been questioned (18, 19) because it is based on digitally scanned graphic data from a single human skin ex vivo sample from one publication (20). Cutaneous previtamin D_3_ and vitamin D_3_ are subject to photochemical degradation, in which case, an action spectrum may change during irradiation (21). Two in vitro action spectra for previtamin D_3_/vitamin D_3_ have been published (22), both of which are blue shifted (i.e., to shorter wavelengths) when compared with the CIE spectrum. A computational model for previtamin D_3_ production in skin, based on an in vitro action spectrum and incorporating human skin’s UVR transmission properties, suggests a blue shift of 2 to 3 nm compared with the CIE action spectrum (23). A study of pig skin in vivo showed peak cutaneous vitamin D_3_ (cholecalciferol) formation at 296 nm (24) that is very similar to the CIE spectrum.

Uncertainty of the CIE previtamin D_3_ action spectrum casts doubt on its validity in risk–benefit calculations. Ideally, in vivo action spectroscopy should be carried out with the gold standard endpoint in vitamin D status, which is serum 25(OH)D_3_, the first hydroxylation product of vitamin D_3_. Conventional action spectroscopy requires monochromatic radiation to generate UVR dose–response curves at different wavelengths for a given endpoint. However, before the recent advent of light-emitting diode sources, the fields of such radiation were typically 4 to 5 mm in diameter and could not be expected to have any significant impact on serum 25(OH)D_3_ and were therefore unsuitable for whole-body studies. It was therefore necessary to take a different approach.

The study goal was to test the hypothesis that the CIE action spectrum for cutaneous previtamin D_3_ predicts UVR-induced increase of serum 25(OH)D_3_ from different UVR spectra. This was approached by generating individual dose–responses for 25(OH)D_3_ from serial exposures to five UVR sources. Exposures were based on SED, after which physical doses (i.e., J/m^2^) were weighted by the previtamin D_3_ CIE action spectrum. The hypothesis would be supported if dose–response data from all spectra fell on the same regression line. A secondary goal was to test for spectral interaction. If such interaction were significant, one would not expect any previtamin D_3_ action spectrum to be valid for a range of UVR sources. Studies were done over partial (<5%) and full (85%) body surface areas to represent adventitious and intentional solar exposure.

## Materials and Methods

The studies were done in London, United Kingdom (51.5° N), during winter/spring of 2011, 2012, 2013, and 2014 when ambient UVB is very low, and skin is protected by clothing.

### Volunteers.

The investigation was done according to the Declaration of Helsinki after ethical approval from the St. Thomas’ Hospital Research Ethics Committee and the National Research Ethics Service Committee, London Bridge, London, United Kingdom. Approval was achieved after detailed submission of the experimental protocol and benefits of the research to the committees that comprises academic, clinical, and lay members. The principal investigator (A.R.Y.) was then questioned in person by board meetings of the committees. Minor amendments/clarifications were requested and made before approval. Normal healthy fair Fitzpatrick skin types (FST) I and II volunteers (*n* = 75) with a mean age of 26.5 ± 5 (SD) years were recruited, the demographic details of whom are shown in Table 1. All volunteers gave written informed consent.

**Table 1.** Demographics of PB and FB studies with baseline, final, and Δ 25(OH)D_3_ values

Area	Spectrum	No.	Gender		Skin type		Age ± SD	Mean 25(OH)D_3_ (nmol/L) ± SD			p
			M	F	I	II		Baseline	Final	Δ	
PB	Fil 2	10	5	5	1	9	26 ± 6	29.3 ± 9.8	41.7 ± 12.3	12.4 ± 7.5	0.00054
	Fil 9	10	3	7	0	10	26 ± 6	37.8 ± 14.7	43.8 ± 15.5	6.0 ± 6.1	0.013
	UV6	15	3	12	3	12	26 ± 4	32.3 ± 13.7	45.8 ± 12.5	13.5 ± 7.8	1.07 × 10^−5^
All PB		35	18	22	4	31	26 ± 5	33.0 ± 13.1	44.0 ± 13.1	11.0 ± 7.8	8.96 × 10^−10^
FB	UV6	10	6	4	2	8	29 ± 6	29.5 ± 17.2	78.5 ± 13.7	49.0 ± 18.3	1.39 × 10^−5^
	Arimed B	19	6	13	0	19	25 ± 3	37.6 ± 21.1	76.1 ± 21.6	38.6 ± 18.4	3.50 × 10^−8^
	PUVA	11	6	5	0	11	26 ± 5	34.3 ± 21.1	54.4 ± 15.0	20.2 ± 12.6	0.00034
All FB		40	18	22	2	38	26 ± 5	34.6 ± 20.0	70.7 ± 20.5	36.1 ± 19.8	3.75 × 10^−14^
All PB + FB		75	29	46	6	69	26 ± 5	33.9 ± 17.0	58.3 ± 21.9	24.4 ± 19.8	1.30 × 10^−16^

*P* values from paired Student’s *t* tests.

### UVR Sources and Dosimetry.

**Fig. 2.**
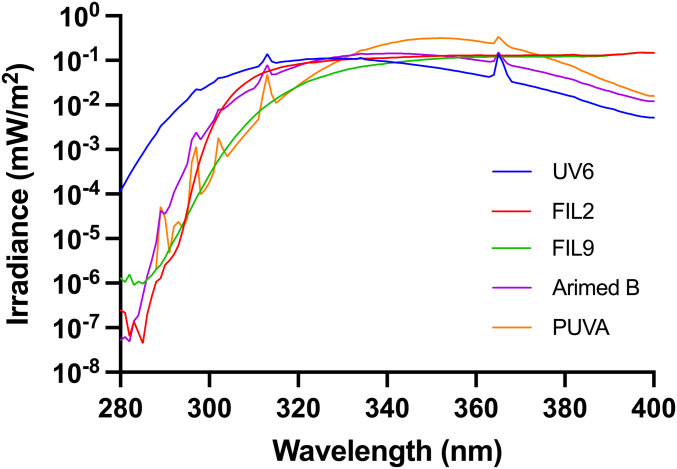
Emission spectra of the 5 UVR sources. The UV6 source was common to both partial and FB studies. The spectrum shown in from the FB unit.

The emission spectra of five UVR sources are shown in Fig. 2, and Table 2 shows their UVB contents as a percentage of each UVR spectrum along with percent EEE. These data show a wide range of UVB:UVA ratios. The irradiance spectra of the sources were determined with a DM150BC double monochromator spectroradiometer (Bentham Instruments) using an integration sphere and gratings blazed at 250 nm and calibrated by the Centre for Radiation, Chemical and Environmental Hazards, Public Health England against a UK national standard. This instrument has a dynamic range of six orders of magnitude. Wavelength calibration was verified using a UVR source with line spectra. The spectroradiometric data were used to calculate the SED exposure times based on weighting with the CIE action spectrum for erythema (7). The spectroradiometric data were also used to calibrate a radiometer (IL 1400, International Light) for day-to-day use.

**Table 2.** Spectral analyses of UVB and UVA content of UVR sources as percent physical quantities and percent EEE

Definition	Analysis	UVR Source									
		UV6		Filter 2		Filter 9		Arimed B		PUVA	
		(PB and FB)		(PB)		(PB)		(FB)		(FB)	
		UVB	UVA	UVB	UVA	UVB	UVA	UVB	UVA	UVB	UVA
CIE	Physical (%)	19.08	80.92	4.52	95.48	0.86	99.14	5.31	94.69	0.90	99.10
(280 to 315 nm)	EEE (%)	95.75	4.25	78.64	21.36	48.38	51.62	79.61	20.39	45.22	54.78
Dermatology	Physical (%)	26.95	73.05	8.05	91.95	2.09	97.91	9.58	90.42	1.68	98.32
(280 to 320 nm)	EEE (%)	97.97	2.03	87.41	12.59	59.65	40.35	87.26	12.74	51.77	48.23
Nonsolar UVB (280 to 295 nm)	Physical (%)	0.97	NA	0.001	NA	0.001	NA	0.02	NA	0.001	NA
	EEE (%)	19.90	NA	0.14	NA	0.81	NA	2.11	NA	0.90	NA
Approx duration of each exposure (s)		59 for PB and 68 for FB		418		1,928		424		1,026	

The wavebands are defined using CIE UVB/UVA boundaries (315 nm) and those more commonly used in photodermatology (320 nm). Note that the UV6 contains 0.97% 280- to 295-nm nonsolar UVB, which contributes to 19.9% of its EEE. Note the variation of duration exposure. NA, not applicable.

There were two studies; one with partial-body (PB) and the other with full-body (FB) exposure. The UVR spectra for the PB study were obtained from a whole-body standup phototherapy unit (Waldmann UV5001 with UV6 tubes, Waldmann GmbH & Co) and a xenon (Xe) arc solar simulator. This was a 1-kW Oriel device (Model 81292, L.O.T Oriel) with two different glass filters (Schott AG) to generate different UVR spectra. Filter 2 was Cosmetics Europe–compliant solar-simulated radiation (SSR) that is used for sunscreen sun protection factor determination. This represents a UVB-rich spectrum that would be found in early December in tropical Australia (∼19° S) (25, 26). Mexico City is ∼19° N with a solar noon midsummer UVB of about 7% of total UVR. Filter 9 has a much lower UVB content and is representative of mid-April UVR in northern latitudes (∼52° N) (26). The body surface area (BSA—Du Bois method) exposed in the PB study (∼3.7%) was 700 cm^2^, which was determined by the limited circular irradiation field (233 cm^2^) of the solar simulator. Exposure was given as two fields on the back and one on the stomach with the solar simulator or one field on the back with the UV6 source.

The whole body, apart from underwear, was exposed in the FB study, which was an estimated 85% BSA. The three spectral sources were Arimed B (Cosmedico) that is a good fluorescent source of SSR, UV6, and PUVA (both Waldmann GmbH & Co). The latter is primarily a UVA source. The tubes were housed in two full-height home phototherapy units (UV 100 L, Waldmann GmbH & Co), each with eight 100-W tubes, arranged as a hexagon to surround the volunteer. The UV6 spectrum was common to both studies, and Table 2 (see caption) shows that ∼1% of nonsolar UVB (280 to 295 nm) contributes ∼20% of its EEE.

### Experimental Protocol.

Each participant received five serial two SED exposures with intervals of 3 to 4 days, resulting in a cumulative dose of 10 SED, which is approximately three MED in the study population. The protocol did not result in erythema. SED were originally calculated with an older version (1987) of the CIE erythema action spectrum. These were therefore recalculated with the updated version (7). Thus, a nominal two SED exposure ranged from 2.02 to 2.45 SED depending on UVR source. Exposure duration varied with spectral source as shown in Table 2.

The physical doses (J/m^2^) for each spectrum were converted into serum 25(OH)D_3_ effective energy (D3EE), where 1 D3EE = 100 J/m^2^ weighted by the CIE previtamin D_3_ action spectrum over a 280 to 400 nm range (14). Analyses of the relationships between D3EE versus 25(OH)D_3_ response, during the course of the serial exposures, were performed to test the validity of the CIE previtamin D_3_ action spectrum for serum 25(OH)D_3_ with the different UVR emission spectra. We tested the hypothesis that a vitamin D_3_-weighed dose would result in a common regression line with all spectra for a given BSA exposure.

Blood samples (∼10 mL) were taken prior to the first exposure and prior to each subsequent exposure (except the second exposure in the FB study) and 3 to 4 days after the last exposure. Blood samples were collected in serum separation tubes and centrifuged at 5,000 × *g* for 10 min. Serum was stored at −80 °C before analysis.

25(OH)D analyses (D_3_ and D_2_) were done by the Department of Clinical Chemistry, Sandwell and Birmingham Hospitals National Health Service (NHS) Trust, Birmingham, United Kingdom, which is a UK Clinical Pathology Accreditation laboratory that participates in the Vitamin D External Quality Assessment Scheme. Samples were assessed by liquid chromatography tandem mass spectrometry (LC-MS/MS) using a Waters Quattro Premier XE Mass Spectrometer attached to a Waters Acquity Ultra Performance LC. All samples from a given volunteer were analyzed in the same run. Duplicate measurements were made in a second run, and the mean was used in the analyses.

### Statistical Analyses.

SPSS Statistics 25 (IBM) was used. ANOVA/multiple linear regressions were employed to determine relationships between UVR dose and 25(OH)D_3_. Normality was assessed by the Kolmogorov–Smirnov test. Linear regression was chosen because there is no evidence of a plateau over the dose range studied. Furthermore, we have previously reported that linear dose–responses provide the best fit on an individual basis with our irradiation protocol (27). Prior to analyses, we also tested the best model fit (R^2^) on an individual basis between 25(OH)D_3_ level and UVR dose in SED (adding 0.1 SED to eliminate zero dose values at baseline) comparing linear, logarithm, inverse, power, and exponential models. We found 28 individuals to have a linear response, 18 to have an exponential response, 10 to follow a power function, one to follow an inverse function, and 18 were nonsignificant. Of the 18 nonsignificant results, 14 (78%) were from the PB exposure study, which also has the lowest increase of 25(OH)D_3_. A linear fit was also the best when the data were pooled for PB and FB exposures (analyzed separately). The linear regression model (which did not have any UVR dose offsets to remove zero dose values) predicts 25(OH)D_3_ after UVR exposure incorporating baseline (prelevel) because this influences 25(OH)D_3_ intercept and steepness of UVR dose response (slope); thus, both effects were included in all models tested as previously published (27, 28). The PB and FB exposures were analyzed separately, using a common model in which the three different UVR sources always were included.

The common linear model has the following form: 25(OH)D (D3EE, UVR source) = intercept (baseline) + slope (baseline, UVR source) × D3EE, where intercept (baseline) = a + b × baseline and slope (baseline) = c × baseline + d (UVR source). Parameter d tests the dependence on UVR source. Independence of UVR source means that an action spectrum can be assumed to be generally valid for all UVR sources. Therefore, the *P* values test of the differences between the different UVR spectra becomes the major outcome of the model. There should be no effect of UVR source if the action spectrum is accurate. The R^2^ values for the whole model are a measure of how well the model fits the observed data for a given exposure (i.e., PB or FB). The best visual representation of the data/model is linear regression of UVR dose versus Δ 25(OH)D_3_ as shown graphically. The significance level was set at *P* < 0.05.

## Results

In most cases, 25(OH)D_2_ values were at the limit of detection. Baseline PB and FB 25(OH)D_3_ were normally distributed with means of 33.0 ± 13.1 (SD) and 34.6 ± 20.00 nmol/L, respectively. The combined normally distributed mean (*n* = 75) was 33.9 ± 17.0 nmol/L. Postexposure 25(OH)D_3_ are shown in Table 1. Model residuals, for individual UVR sources and combined, were normally distributed (*P* > 0.055 for Arimed B and > 0.18 for other spectra).

Dose weighting with the CIE erythema action spectrum showed that the regression slopes of the different spectra, within PB and FB experiments, were significantly different (*P* < 0.005). This was also the case (*P* < 0.005) with weighting with the CIE action spectrum for previtamin D_3_ (14) and therefore did not result in common regression lines for the different UVR sources.

**Fig. 3.**
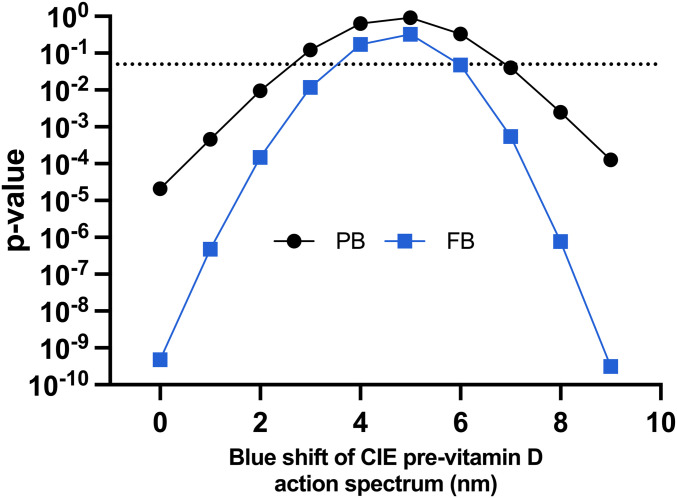
Relationship between *P* value and blue shift of CIE previtamin D_3_ action spectrum for previtamin D_3_. The shifts above the *P* = 0.05 dotted line show no significant differences between the dose–response slopes for the different UVR spectra. That is, the optimized action spectrum works as a weighting function irrespective of UVR source. The highest *P* values are with a 5-nm shift for both PB and FB exposures. The data clearly show that the CIE spectrum (zero shift) is inadequate.

There are two in vitro previtamin D_3_/vitamin D_3_ action spectra in the literature (29, 30) that show a blue shift compared with the CIE spectrum (31). The Bolsee spectrum (29) worked well for PB exposure, but there was a just significant difference (*P* = 0.049) in slopes of the UVR sources for FB exposure. However, the Olds spectrum (30) showed highly significant differences in slopes of the UVR sources for the PB and FB studies. We then tested the effect of incremental 1-nm blue shifts up to 9 nm with the CIE action spectrum for previtamin D_3_ without changing the shape of CIE action spectrum, the results of which are shown in Table 3. The relationship between blue shift and *P* is shown in Fig. 3. The difference between the slopes of the UVR sources was lost at 3 nm for the PB studies (*P* = 0.123) and 4 nm for the FB studies (*P* = 0.171). The highest *P* values were seen with a 5-nm shift for PB (*P* = 0.911) and FB (*P* = 0.326) studies. The 5-nm blue shift adjusted action spectrum is shown in Fig. 1 along with the original CIE spectrum. This difference was recovered (*P* < 0.05) with shifts ≥7 nm for PB and shifts ≥6 nm for FP. The nonsignificant range of shifts was 3 to 6 nm for PB and 4 to 5 nm for FB that gives an indication of the error in our model. That this range was tighter with FB exposure is not surprising because the 25(OH)D_3_ response was greater. The 5-nm shift also resulted in the best R^2^ value for the model with common slopes for the different UVR sources with the PB and FB studies. Thus, the results described below are for a 5-nm shift.

**Table 3.** Model linear regression parameters, after baseline adjustment, for 25(OH)D_3_ versus UVR dose weighted with different action spectra for PB and FB exposures

Action spectrum 1 nm = 1 nm blue shift (toward shorter wavelengths)	PB exposure		FB exposure	
	*P* value for differences between three spectral slopes	R^2^ value for model with common slope	*P* value for differences between three spectral slopes	R^2^ value for model with common slope
CIE erythema	<0.0005	0.800	<0.0005	0.770
CIE previt D	<0.0005	0.802	<0.0005	0.805
CIE previt D 1 nm	<0.0005	0.809	<0.0005	0.819
CIE previt D 2 nm	0.009	0.816	<0.0005	0.831
CIE previt D 3 nm	0.123	0.822	0.012	0.839
CIE previt D 4 nm	0.640	0.826	0.171	0.845
CIE previt D 5 nm	0.911	0.828	0.326	0.847
CIE previt D 6 nm	0.330	0.826	0.047	0.845
CIE previt D 7 nm	0.040	0.823	0.0005	0.838
CIE previt D 8 nm	0.002	0.818	<0.0005	0.828
CIE previt D 9 nm	<0.0005	0.813	<0.0005	0.814
Bolsee (29)	0.841	0.827	0.049	0.843
Olds (30)	0.004	0.819	<0.0005	0.834

The *P* values test the differences between the different UVR spectra. There should be no effect of UVR source if the action spectrum is accurate. The R^2^ values are for the whole model for a given exposure (i.e., PB or FB) protocol. The data show that the highest *P* values for lack of differences between the slopes of the three spectra used in each study condition occur with a 5-nm blue shift of the CIE previtamin D action spectrum. This also results in the highest R^2^ values for the common slopes. The PB data fit an in vitro previtamin D_3_ spectrum (29), though the difference between the three slopes of FB data is borderline significant but not an in vitro spectrum for vitamin D_3_ (cholecalciferol) (30).

### PB Study.

**Fig. 4.**
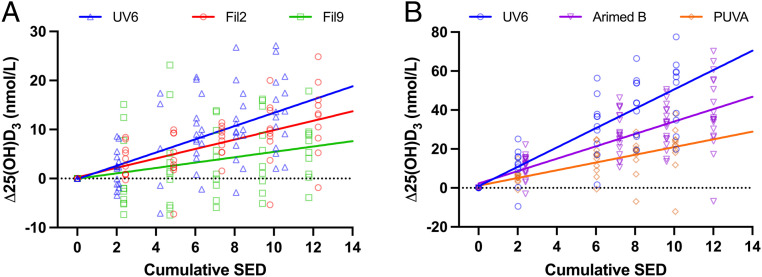
Linear regressions (with no baseline correction) for Δ 25(OH)D_3_ versus SED for studies with (*A*) PB and (*B*) FB exposures. Regression parameters, not forced through zero, for (*A*) PB: UV6 y = 1.35x – 0.09, *R*^2^ = 0.41, *P* = 1.67 × 10^−10^; filter 2 y = 0.96x + 0.26, *R*^2^ = 0.42, *P* = 2.31 × 10^−8^; filter 9 y = 0.55x + 0.04, *R*^2^ = 0.09, *P* = 0.0177 and (*B*) FB UV6 y = 4.97x + 0.86, *R*^2^ = 0.68, *P* = 1.78 × 10^−13^; Arimed B y = 3.18x + 2.26, *R*^2^ = 0.63, *P* = 1.09 × 10^−21^ and PUVA y = 1.99x + 1.12, *R*^2^ = 0.47, *P* = 6.29 × 10^−9^. All intercepts were not significantly different from zero, *P* > 0.25.

**Fig. 5.**
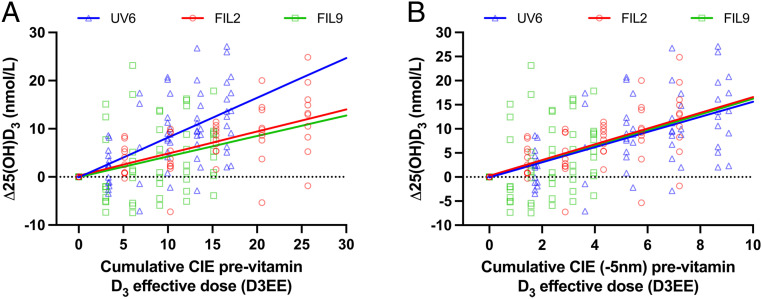
PB linear regressions (with no baseline correction) for Δ 25(OH)D_3_ versus UVR dose weighted with CIE previtamin D action spectrum with (*A*) no shift or (*B*) with 5-nm blue shift. Regression parameters, not forced through zero, for (*A*) no shift: UV6 y = 0.8x − 0.1, filter 2 y = 0.5x + 0.26, filter 9 y = 0.4x − 0.04 and for (*B*) 5-nm shift: UV6 y = 1.6x − 0.09, filter 2 y = 1.6x + 0.26, filter 9 y = 1.6x − 0.04. All R^2^ and *P* values for a given spectrum as for Fig. 4*A*. The common model for 25(OH)D, including baseline correction: 25(OH)D (in nmol/L) = −1.31[1.83] (*P* = 0.47) + 1.04[0.051] (*P* = 0.38 compared to 1) × pre-25(OH)D − 0.033[0.012] (*P* = 0.0046) × pre-25(OH)D × D3EE + 2.64[0.41] (*P* = 6.75 × 10^−10^) × D3EE, [SD]. 1 D3EE = 100J/m^2^ weighted by the CIE previtamin D_3_ action spectrum with or without 5-nm blue shift.

There was a linear relationship (*P* = 1.36 × 10^−9^) between pre- and post-25(OH)D_3_ with a slope of 0.82 (i.e., close to 1) as previously reported (32). Thus, there is a lowered postexposure increase of 0.18 (1 − 0.82) nmol/L per pre-nmol/L 25(OH)D_3_, which confirms the importance of incorporating the prevalue into the regression model. This information would be lost in a model based only on the difference (Δ) between post- and prevalues, especially when increased 25(OH)D_3_ with <5% BSA exposed was relatively small (final Δ 25(OH)D_3_ = 11.00 ± 7.8 [SD] nmol/L for all studies combined). For illustrative purposes, we have used the Δ 25(OH)D values (without baseline correction) in the linear regression figures. Fig. 4*A* shows the relationship between SED and Δ25(OH)D_3_. All UVR source model slopes were highly significant with UV6 (*P* = 1.67 × 10^−7^) > filter 2 (*P* = 2.12 × 10^−7^) > filter 9 (*P* = 0.00375) with *P* values determined in a common model. Fig. 5*A* shows the effect of weighting UVR dose with the CIE action spectrum for previtamin D_3_. The model shows no difference between two solar spectra slopes (*P* = 0.865), which, when combined, was significantly less steep (*P* = 3.40 × 10^−6^) than the UV6 slope. Thus, the CIE previtamin D_3_ action spectrum is valid for two very different solar-simulated spectra (one with low SZA [filter 2] and one with high [filter 9]) but underestimates synthesis with nonsolar UVB radiation. However, Fig. 5*B* shows that a 5-nm blue shift of the CIE previtamin D_3_ action spectrum results in virtually identical dose–response curves for all three spectra, and this is supported by the common model as shown in Table 3 (*P* = 0.911 for overall slope difference, and *P* > 0.675 for individual slope comparisons). The model shows that 1 D3EE increases serum 25(OH)D_3_ by 1.6 nmol/L with a baseline 25(OH)D_3_ value of 33.0 nmol/L. The 25(OH)D_3_ increase is raised by 1.09 nmol/L to a maximal increase of 2.6 nmol/L with zero baseline value. The calculation of observed power for the 5-nm blue-shifted model gave a value of 0.999. This model also showed no effect of age or sex on intercept (*P* > 0.44) or slope (*P* ≥ 0.20).

### FB Study.

**Fig. 6.**
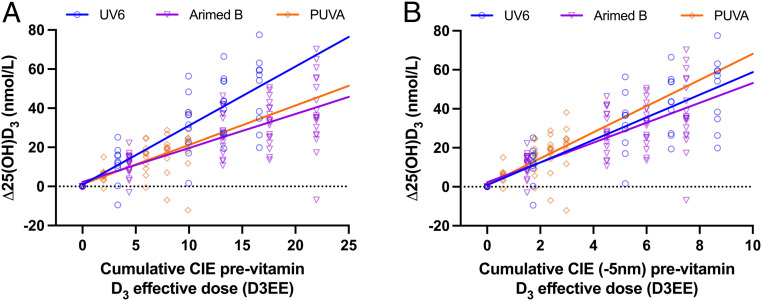
FB linear regressions (with no baseline correction) for Δ 25(OH)D_3_ versus UVR dose weighted with CIE previtamin D action spectrum with (*A*) no shift or (*B*) 5-nm blue shift. Regression parameters, not forced through zero, for (*A*) no shift: UV6 y = 3.0x + 0.86, Arimed B y = 1.7x + 2.26, and PUVA y = 2.0x + 1.1 for (*B*) 5-nm shift: UV6 y = 5.8x + 0.86, Arimed B y = 5.1x + 2.26, and PUVA y = 6.7x + 1.12. All R^2^ and *P* values for a given spectrum as for Fig. 4*B*. The common model including baseline correction: 25(OH)D (in nmol/L) = 4.05[2.04] (*P* = 0.049) + 0.95[0.05] (*P* = 0.32 compared to 1) × pre-25(OH)D − 0.059[0.012] (*P* = 1.54 × 10^−6^) × pre-25(OH)D × D3EE + 7.35[0.46] (*P* = 6.95 × 10^−37^) × D3EE, [SD]. 1 D3EE = 100J/m^2^ weighted by the CIE previtamin D_3_ action spectrum with or without 5-nm blue shift.

There was a linear relationship (*P* = 0.00053) between pre- and post-25(OH)D_3_ with a slope of 0.54. The presentational and analytical approaches are as described above. Fig. 4*B* shows the relationship between SED and Δ25(OH)D_3_. The model slopes were highly significant with UV6 (*P* = 1.244 × 10^−38^) > Arimed B (*P* = 2.851 × 10^−31^) > PUVA (*P* = 9.215 × 10^−16^). There is significant difference between comparisons of any two of the three slopes of Arimed B, PUVA, and UV6 (*P* ≤ 2.078 × 10^−7^). Fig. 6*A* shows the relationship between Δ25(OH)D and UVR dose weighted with the previtamin D_3_ CIE action spectrum. There is no difference between the model slopes of Arimed B and PUVA (*P* = 0.675), but these are significantly less steep than for UV6 (*P* ≤ 0.00025). Fig. 6*B* shows the effect of weighting the UVR doses with the CIE previtamin D_3_ action spectrum with a 5-nm blue shift, and this is supported by the common model (Table 3) with no significant difference between any two slopes of any of the three UVR sources (*P* = 0.326 for overall slope difference, and *P* > 0.139 for individual slope comparisons). The model shows that 1 D3EE increases serum 25(OH)D_3_ by 5.3 nmol/L with a baseline 25(OH)D_3_ value of 34.6 nmol/L. The 25(OH)D_3_ increase is raised by 2.0 nmol/L to a maximum of 7.3 nmol/L with a baseline value of zero. The calculation of observed power for the 5-nm blue-shifted model gave a value of 1.0. This model also showed no effect of age or sex on intercept (*P* ≥ 0.20) or slope (*P* ≥ 0.10).

## Discussion

Pooled baseline (i.e., pre) 25(OH)D_3_ of all 75 participants was normally distributed. The mean value of 33.87 nmol/L ± 17.00 (SD) is lower than that reported for winter in UK adults (18 to 69 y old) (33) and adolescents (12 to 15 y old) (34). We demonstrated a significant relationship between pre- and post-25(OH)D_3_ in which the higher the baseline, the lesser the response to UVR. This supports our previously reported laboratory (32) and field studies (28) and confirms the importance of including baseline 25(OH)D_3_ in the regression model. Baseline 25(OH)D_3_ values were essentially the same in the PB and FB studies, but interestingly, the effect of baseline on 25(OH)D_3_ increase was greater for FB (85% BSA exposed) than PB (3.7% BSA exposed) and raises the possibility of an influence of BSA exposed. Some studies of longer duration than our study have shown a linear increase of 25(OH)D_3_ with increasing cumulative UVR exposure followed by a plateau (35, 36). We tested various models and found a linear UVR dose response to be the best fit for both individual and pooled PB and FB data over the 2- to 3-wk duration of the studies. It should be noted that there was considerable interpersonal variation in the results, but this is a common feature in similar studies, and one likely reason is genetic differences (37). Unless replenished, 25(OH)D_3_ decreases with time. We had no controls (i.e., nonirradiated) in this study, but there were such controls in another study during the same time period with a very similar irradiation protocol (27). Control participants showed no significant change of serum 25(OH)D_3_ during the study period. We also reported no significant change in vitamin D status in control volunteers over 1 wk in another study (38). We therefore do not think catabolism of 25(OH)D_3_ had any impact on our results.

SED was used to measure exposure because erythema is a widespread clinical endpoint, and this approach reduced the likelihood of inadvertent erythema. Furthermore, the SED is used in population exposure studies (39, 40). As expected, the greater the UVB contribution to erythema (Table 2), the greater the effectiveness at vitamin D_3_ production with exposure expressed as SED (Fig. 4). Thus, our results confirm that the SED is a poor predictor of vitamin D_3_ synthesis, despite the apparent similarity of the action spectra for erythema and previtamin D_3_ (whether adjusted or not) in the UVB region. This is because wavelengths >330 nm contribute to erythema but not vitamin D_3_ production, based on CIE action spectra.

The validity of the CIE previtamin D_3_ action spectrum was tested as a surrogate for the action spectrum for serum 25(OH)D_3_ with five different broadband UVR spectra; two of which represent solar spectral extremes. The working hypothesis was that the previtamin D weighted dose–response curves for 25(OH)D_3_ would fall on the same regression line if the CIE action spectrum for previtamin D_3_ were applicable for 25(OH)D_3_. Figs. 5*A* and 6*A* show that this was not the case for PB and FB exposures, respectively. Possible reasons for this discrepancy include 1) action spectra for cutaneous previtamin D_3_ and serum 25(OH)D_3_ are different, 2) there is spectral interaction (e.g., photodegradation) in the formation of vitamin D_3_ such that action spectroscopy cannot predict outcome from broadband sources, or 3) there is an error in the action spectrum for previtamin D_3_. The latter option was tested by iteration with progressive 1-nm blue shifts of the CIE action spectrum because two in vitro action spectra for previtamin/vitamin D_3_ synthesis show peak blue shifts of about 5 nm (22), and recent calculations have also suggested that the CIE action spectrum should be blue shifted (21, 23). Table 3 shows the best fit, for PB and FB studies, was with a 5-nm blue shift of the CIE action spectrum, although shifts of 3 to 6 nm and 4 to 5 nm may also be valid for PB and FB, respectively. This shift is also a better match for the absorption spectrum peak of 7-DHC (21) that is present in all epidermal layers but at higher concentrations in the upper epidermis (23, 41); the higher the chromophore in the epidermis, the less the impact of competing chromophores. Our PB and FB data did not fit the Olds action spectrum (30) for vitamin D_3_ in vitro (measured 24 h after exposure to allow previtamin D_3_ to thermally convert to vitamin D_3_), but the PB data fitted the Bolsee action spectrum (29) for previtamin D_3_ in vitro, and there was a borderline fit for the FB data. The lack of fit with the Olds spectrum is not surprising because irradiation of 7-DHC in a quartz vessel lacks the attenuating effects of skin, which are stronger at the shorter wavelengths. However, the same considerations would apply to the Bolsee spectrum. Our data are not consistent with a study in which 7-DHC was added to a human skin equivalent model and peaks were found at ∼302 nm for calcitriol (1α,25(OH)_2_D_3_) and vitamin D_3_ (42). This is a red shift compared to the peak of 297 nm from the action spectrum for previtamin D_3_ in ex vivo human skin (20) on which the CIE action spectrum is based. The reasons for these differences are not known (18), but one possibility is greater UVR attenuation by the skin model. Indeed, it has been suggested that vitamin D action spectra may vary with body site because of differences in epidermal transmission (21). Very limited information was given on the spectral characteristics of the monochromatic emission spectra (20) on which the CIE previtamin D_3_ spectrum is based. Thus, is it possible that these spectra had biologically significant quantities of shorter wavelength UVR. It is very important to have spectral measurements with a very large dynamic range (e.g., six orders of magnitude) to measure small amounts of “contaminating” radiation in a given spectrum. For example, 0.8% UVB in a UVA tanning lamp caused 75% of the DNA damage in keratinocytes in vitro (43).

One in vitro study reported that UVAII (315/320 to 340 nm) degrades vitamin D_3_ as part of a homeostatic mechanism (44). However, we suggest that biologically significant spectral interaction is unlikely in vivo given the wide range of ratios of UVA to UVB in the study spectra (Table 2). The spectra with the greatest relative UVA content (filter 9 [PB] and PUVA [FB]) also comply with a 5-nm shift as well as the Bolsee spectrum (29) for PB exposure. We therefore propose a 5-nm blue shift for the CIE spectrum based on our data, as shown in Fig. 1, especially because they apply to serum 25(OH)D_3_, which is used to assess vitamin D status.

Fig. 5*A* shows that the CIE action spectrum (with no shift) for the synthesis of previtamin D is valid, with two solar spectra representing extremes of UVB exposure (summer at an equatorial latitude and winter at a northern latitude). This supports the use of the CIE spectrum with solar spectra in risk–benefit calculations of health outcomes. However, Fig. 5*A* also indicates that the CIE spectrum is not valid for a source that contains nonsolar UVB, which means that this spectrum results in more vitamin D_3_ than predicted. This is important because many studies have used such spectra. However, a 5-nm blue shift of the CIE spectrum (Fig. 5*B*) readily incorporates all three spectra. In the PB studies, Fig. 5*A* also shows the CIE action spectrum results in more 25(OH)D_3_ than expected with UV6 that contains the greatest proportion of nonsolar short-wave UVB (physically and EEE). Fig. 5*B* shows that a 5-nm blue shift readily incorporates all three spectra. Comparisons of Figs. 5*B* and 6*B* show better results with the PB exposures. One possible reason is that the PB exposures were limited to back and stomach. The action spectrum is modified by the optical properties of the skin that vary considerably with body site (21, 23). Thus, the FB exposures may reflect a composite action spectrum.

Filter 9 and PUVA with UVB/UVA EEE of ∼50/50 is representative of mid-April noon London 51.5° N (26). It is often stated, based on studies with solutions of 7-DHC and excised skin, that vitamin D_3_ synthesis cannot occur during the “vitamin D winter,” which, for example, is from November to February at 42° N (Boston) and from October to April at 52° N (Edmonton) (45). Our in vivo data support vitamin D synthesis in London, which is comparable in latitude (51.5° N) to Edmonton, in mid-April because the regression slope of 25(OH)D_3_ with filter 9 (PB) was highly significant with regressions against dose weighted for erythema and previtamin D with and without the 5-nm adjustment. This was also the case for PUVA (FB). It should be noted that the concept of the “vitamin D winter” has been queried (46).

McKenzie et al. (46), with a very different protocol from our study (e.g., two UVR spectra, 12-wk duration, three blood samples, and radioimmunoassay for 25(OH)D_3_), reported that their results showed “no obvious inconsistency in the action spectrum (CIE) for previtamin D production.” However, in a later, more comprehensive study (22), the same group reported that vitamin D action spectra (CIE, Bolsee, and Olds) did not perform any better than the CIE erythema action spectrum in estimating increases in 25(OH)D_3_, which indicates that they are inadequate.

The study strengths are the large sample size, a range of five different UVR spectra and serum 25(OH)D_3_, determined by LC-MS/MS by an accredited laboratory, as the endpoint. It was also done with suberythemal exposure, which is the safest way to improve vitamin D status in the sun (47). Different results may have been obtained with high-dose erythemal exposures, but such doses would have been neither ethical nor consistent with public health advice. There was also considerable interpersonal variation within a given UVR exposure protocol, which was almost certainly due to genetic factors (37). A weakness is the lack of data on melanized skin types because photobiological responses are affected by skin type (48–51). The quantitative impact of melanin on vitamin D synthesis remains controversial with recent studies casting doubt on the need for vitamin D as a driver for the evolution of light skin with *Homo sapiens*’ migration from Africa (52, 53). The results from this study only apply to FST I/II, and it will be important to assess the effect of skin melanin on the action spectrum for 25(OH)D_3_. It should be noted that there was a wide range of irradiation times, which was a technical limitation. However, dose rather than dose rate (irradiance) is the main factor for serum increase in 25(OH)D_3_ (54).

In conclusion, SED is a poor predictor of vitamin D synthesis. It should be noted that SED can be readily used as a standard because erythema is localized. In contrast, D3EE refers to a systemic outcome, that is, increase of 25(OH)D_3_ that depends on area of body irradiated, for a given UVR dose protocol. The current CIE previtamin D_3_ action spectrum is valid for 25(OH)D_3_ induced by two diverse solar-simulated sources but not for sources containing nonsolar UVB, which are widely used in laboratory studies. Thus, published risk/benefit solar UVR calculations using the CIE action spectra for erythema and previtamin D_3_ are likely to be valid, though this remains to be tested. Nonetheless, an improved action spectrum based on serum 25(OH)D_3_, rather than cutaneous previtamin D_3_, will result in more refined assessments of risks and benefits of solar exposure that should result in better public health advice. In contrast, the CIE previtamin D_3_ action spectrum is not valid when using broad-spectrum sources with nonsolar UVB wavelengths. In two studies with different spectra and irradiation fields, we show that a 5-nm blue shift of the CIE previtamin D_3_ action spectrum provides a better model for the synthesis of 25(OH)D_3_ than the current CIE action spectrum. Our proposed blue shift also provides a better match with the absorption spectrum peak of 7-DHC. The ability to fit all data with a single action spectrum does not support significant spectral interaction for the synthesis of 25(OH)D_3_. Our data also show that regular suberythemal solar UVR exposure to ∼4% BSA improves vitamin D status. More research is needed to determine optimal BSA and UVR dose to gain the maximum benefits from solar exposure while mitigating the risks.

## Data Availability

Anonymized data in Excel format have been deposited in Open Science Framework (https://osf.io/xw467/) (55).
